# Interactions among morphotype, nutrition, and temperature impact fitness of an invasive fly

**DOI:** 10.1002/ece3.4928

**Published:** 2019-02-03

**Authors:** Dalila Rendon, Vaughn Walton, Gabriella Tait, Jessica Buser, Ivana Lemos Souza, Anna Wallingford, Greg Loeb, Jana Lee

**Affiliations:** ^1^ Department of Horticulture Oregon State University Corvallis Oregon; ^2^ Research and Innovation Centre Fondazione Edmund Mach San Michele all'Adige Italy; ^3^ Department of Entomology Federal University of Lavras Lavras Brazil; ^4^ Department of Entomology Cornell University Geneva New York; ^5^ University of New Hampshire, Cooperative Extension Durham New Hampshire; ^6^ USDA ARS Horticultural Crops Research Unit Corvallis Oregon

**Keywords:** carbohydrates, *Drosophila suzukii*, fecundity, lifespan, overwintering, protein

## Abstract

Invasive animals depend on finding a balanced nutritional intake to colonize, survive, and reproduce in new environments. This can be especially challenging during situations of fluctuating cold temperatures and food scarcity, but phenotypic plasticity may offer an adaptive advantage during these periods. We examined how lifespan, fecundity, pre‐oviposition periods, and body nutrient contents were affected by dietary protein and carbohydrate (P:C) ratios at variable low temperatures in two morphs (winter morphs WM and summer morphs SM) of an invasive fly, *Drosophila suzukii.* The experimental conditions simulated early spring after overwintering and autumn, crucial periods for survival. At lower temperatures, post‐overwintering WM lived longer on carbohydrate‐only diets and had higher fecundity on low‐protein diets, but there was no difference in lifespan or fecundity among diets for SM. As temperatures increased, low‐protein diets resulted in higher fecundity without compromising lifespan, while high‐protein diets reduced lifespan and fecundity for both WM and SM. Both SM and WM receiving high‐protein diets had lower sugar, lipid, and glycogen (but similar protein) body contents compared to flies receiving low‐protein and carbohydrate‐only diets. This suggests that flies spend energy excreting excess dietary protein, thereby affecting lifespan and fecundity. Despite having to recover from nutrient depletion after an overwintering period, WM exhibited longer lifespan and higher fecundity than SM in favorable diets and temperatures. WM exposed to favorable low‐protein diet had higher body sugar, lipid, and protein body contents than SM, which is possibly linked to better performance. Although protein is essential for oogenesis, WM and SM flies receiving low‐protein diets did not have shorter pre‐oviposition periods compared to flies on carbohydrate‐only diets. Finding adequate carbohydrate sources to compensate protein intake is essential for the successful persistence of *D. suzukii* WM and SM populations during suboptimal temperatures.

## 1 INTRODUCTION

Invasive species commonly encounter temperature and diet fluctuations as they colonize and establish in new environments. Due to their economic and health impact, great attention has been given lately to the biology and ecology of invasive insects (Beukeboom, 2018; Garnas et al., 2016). Multiple mechanisms can be attributed to the success of invasive insects in challenging conditions. First, many polyphagous invasive species are able to exploit multiple food resources to acquire nutrients in new environments (Leclaire & Brandl, 1994). Second, phenotypic plasticity allows organisms to develop characteristics that help them adapt to variable conditions (Fordyce, 2006; Moczek, 2010). Third, invasive species have physiological and behavioral adaptations that allow them to survive through bottleneck periods, such as winter. Interactions among these mechanisms contribute to the overall fitness of these organisms as they disperse and colonize new environments.

Nutritional balance is essential for animal survival and reproduction (Grandison, 2009; Simpson & Raubenheimer, 2011). As a result, there is interest in exploring how macronutrient intake affects various fitness parameters in invasive insects, such as the spotted‐wing drosophila *Drosophila suzukii* Matsumura (Diptera: Drosophilidae; Jaramillo, Mehlferber, & Moore, 2015; Plantamp, Estragnat, Fellous, Desouhant, & Gibert, 2017; Silva‐Soares, Nogueira‐Alves, Beldade, & Mirth, 2017; Tochen, Walton, & Lee, 2016), the emerald ash borer *Agrilus planipennis* (Coleoptera: Buprestidae; Chen, Ciaramitaro, & Poland, 2011), and the Argentine ant *Linepithema humile* (Hymenoptera: Dolichoderinae; Kay, Zumbusch, Heinen, Marsh, & Holway, 2010). An adequate balance of essential macronutrients has important fitness implications. In particular, the protein: carbohydrate (P:C) ratio in diet influences lifespan and fecundity in many insect taxa (Le Couteur et al., 2016; Fanson & Taylor, 2012; Lee, 2015; Rho & Lee, 2016). *Drosophila melanogaster* regulates its sugar, yeast, and water intake (Fanson, Yap, & Taylor, 2012), presumably because dietary P:C ratios are known to play an important role on the survival and fecundity of drosophilid flies and tephritid fly adults. Specifically, high‐protein and low‐carbohydrate diets can reduce lifespan in adult *D. melanogaster* (Bruce et al., 2013; Jensen, McClure, Priest, & Hunt, 2015; Lee, 2015; Ponton et al., 2015) and the tephritid *Batrocera tryoni* (Fanson & Taylor, 2012). As insects disperse and colonize new environments, a balanced nutrient intake is essential for their survival and persistence.

Environmental stressors, temperature fluctuations, and physiological needs can determine how macronutrients are allocated in the body. Depending on the need, carbohydrates can be immediately used for energy, transformed into lipids for storage, or used in somatic maintenance, while proteins are essential for reproduction (Le Couteur et al., 2016). Decreasing ambient temperatures can signal the need for reducing metabolic rate and investing in energy storage to prepare for winter. For instance, in mammals, low temperatures can result in a reduction in body fat (Landsberg, 2012), while in vinegar flies, low temperatures can increase lifespan (Conti, 2008). Additionally, cold temperatures can enhance the lifespan benefits of low‐protein, high‐carbohydrate diets in some taxa (Le Couteur et al., 2016). Similarly, increasing temperatures after winter may trigger production of eggs and energy investment in reproduction (Sinclair, 2015). As such, variable temperatures may have an effect on the optimal nutrient balance and body composition of an organism; yet, the interactive or synergistic effects of diet and temperature on lifespan and fecundity in many taxa are not well understood.

As invasive insects disperse into higher latitudes, they may experience longer periods of suboptimal temperatures and limited resources, which can affect their survival and reproductive potential. Many temperate insects halt reproduction at low temperatures during winter months (Allen, 2007), but readily resume egg maturation and oviposition as temperatures increase (Grassi et al., 2018; Lehmann, Bijl, Nylin, Wheat, & Gotthard, 2017; Ryan, Emiljanowicz, Wilkinson, Kornya, & Newman, 2016; Toxopeus, Jakobs, Ferguson, Gariepy, & Sinclair, 2016; Wallingford, Lee, & Loeb, 2016; Wallingford & Loeb, 2016). When temperatures increase and decrease during spring and autumn, organisms may encounter a change in the availability of food resources with different nutritional composition (Irwin, Raharison, Raubenheimer, Chapman, & Rothman, 2015). In temperate organisms, epigenetics may offer an adaptive strategy to variable environments (Burggren, 2017), as various phenotypes (i.e., summer morphs [SM] or winter morphs [WM]) arise depending on developmental temperatures, making them better suited to withstand challenging conditions relative to the season (Fraimout et al., 2018; Shearer et al., 2016; Wallingford & Loeb, 2016). Fluctuation in resources between seasons may cause differences in how SM and WM allocate their nutrients; for example, WM metabolism could be optimized toward storage and migration, rather than reproduction, until conditions and resources become more favorable to resume oviposition. The interaction between dietary macronutrient intake and variable temperatures likely impacts survival and reproduction in insects, but these effects are usually studied in a single morphotype.

In this study, we explore the interactions among nutrition, environmental stressors, and phenotype in an economically relevant invasive species, the spotted‐wing drosophila *Drosophila suzukii* Matsumura (Diptera: Drosophilidae). *Drosophila suzukii* originated in temperate Asia and has successfully invaded North America, South America, and Europe since its first detection outside its native range in 2008 (Dos Santos et al., 2017; Lee et al., 2011; Walsh et al., 2011). This agricultural pest poses a threat for berry and cherry production, as females possess a serrated ovipositor which enables them to lay eggs inside ripening fruit (Karageorgi et al., 2017). The rapid spread and establishment of *D. suzukii* can be attributed, in part, to the ability of both WM and SM phenotypes to exploit multiple fruit hosts for oviposition (Grassi et al., 2018; Kenis et al., 2016; Lee et al., 2015), their resilience to colonize new environments, and their adaptability to harsh environmental conditions (Stockton, Wallingford, & Loeb, 2018). There is evidence that SM larvae and pupae do not survive cold temperatures (Dalton et al., 2011; Stockton et al., 2018) and that *D. suzukii* overwinters in temperate regions as adult WM (Rossi‐Stacconi et al., 2016). As such, the nutritional balance of WM adults during changing environments is essential for their permanence and dispersal.

We here tested fitness parameters and nutritional profiles of adult WM and SM *D. suzukii* receiving diets with variable P:C ratios under suboptimal temperatures (where reproduction decreases, Ryan et al., 2016). Specifically, we ask whether (a) WM have longer lifespans and higher fecundity than SM at lower temperatures, (b) if WM and SM have different optimal P:C requirements for lifespan and reproduction, and (c) if WM and SM have different body nutrient profiles that may explain their dietary requirements at suboptimal temperatures. The results of this study provide further insight on the physiological adaptations of invasive insects in new and variable environments.

## 2 MATERIALS AND METHODS

### 2.1 Fly rearing

*Drosophila suzukii* used in the rearing of WM and SM came from a laboratory colony maintained at the Horticultural Crops Research Unit, United States Department of Agriculture–Agricultural Research Service (USDA‐ARS) in Corvallis, Oregon. For detailed fly‐rearing protocols, see Rendon, Buser, Tait, Lee, and Walton (2018). Briefly, WM flies were reared by placing cornmeal diet dishes with eggs in a controlled environment chamber (14°C, 12L:12D). Upon emergence (approximately a month later), groups of 50 flies were transferred to 236 ml rearing bottles and provided with cornmeal agar. After 8–10 days, females were separated in groups of 20–25 and transferred to a “cold‐hardening” chamber (7°C, 12L:12D) for one week. After cold hardening, females were transferred to a simulated overwintering cold room (1°C, 8L:16D) for five weeks. Previous work showed that five weeks in these conditions are enough to cause significant, but not total mortality (Wallingford, Rice, Leskey, & Loeb, 2018). Females were offered cornmeal diet during the first week of overwintering and an agar diet during the remaining four weeks (32 g LB agar + 1 L dH_2_O; to simulate food resource depletion).

After the overwintering period, surviving females were individually paired with a 3‐ to 4‐week‐old mature WM male (kept at 14°C) in a rearing bottle and randomly assigned to different diet and temperature treatments (see below). Approximately 2 ml of diet were poured in a 35‐mm petri dish placed at the base of the rearing bottle, where flies could freely feed and oviposit. The percent of surviving flies in each date cohort at the end of the 5‐week overwintering period was recorded.

SM flies were reared by incubating the eggs in a walk‐in colony room (22°C, 16L:8D). Upon emergence, groups of 50 flies were placed in rearing bottles (as described for WM) and offered a sugar agar diet (130 g sucrose, 32 g agar, 1 L dH_2_O) for 24 hr. After 24 hr, one male and one female were transferred to a new rearing bottle and randomly assigned to different diet and temperature treatments.

### 2.2 Diet and temperature treatments

We prepared agar diets varying in protein (P) to carbohydrate (C) ratios (P:C 0:0, 0:1, 1:4, 1:2, 1:1, in increasing protein order), following previously described formulations that affect *Drosophila* lifespan and fecundity (Lee, 2008; Ponton et al., 2015). Agar diets were prepared with sucrose and yeast hydrolysate (45% protein, 24% carbohydrate, 21% indigestible fiber, 8% water, 2% fatty acids, minerals, and vitamins; #103304, MP Biomedicals, Santa Ana, CA, USA), using yeast hydrolysate (Y) to sucrose (S) ratios of 0:0, 0:1, 1:1.6, 1:0.7, and 1:0.2, respectively, to obtain the appropriate P:C ratios. Each diet contained a total of 180 g Y + S, and 32 g LB agar (#22700025, Thermo Fisher Scientific, Waltham, MA, USA) in 1 L dH_2_O, and 3.7 ml 1 M propionic acid, 0.69 g methylparaben, and 6.9 ml 95% ethanol as anti‐mold agents.

Based on temperatures that are suboptimal for *D. suzukii* reproduction (Ryan et al., 2016; Tochen et al., 2014), we set up five temperature treatments in separate controlled‐environment chambers (7, 9, 12, 14, and 17°C, 12L:12D photoperiod, 200 Lux; LED 30HL1, Percival Scientific Inc., Perry, IA, USA).

**Figure 1:**
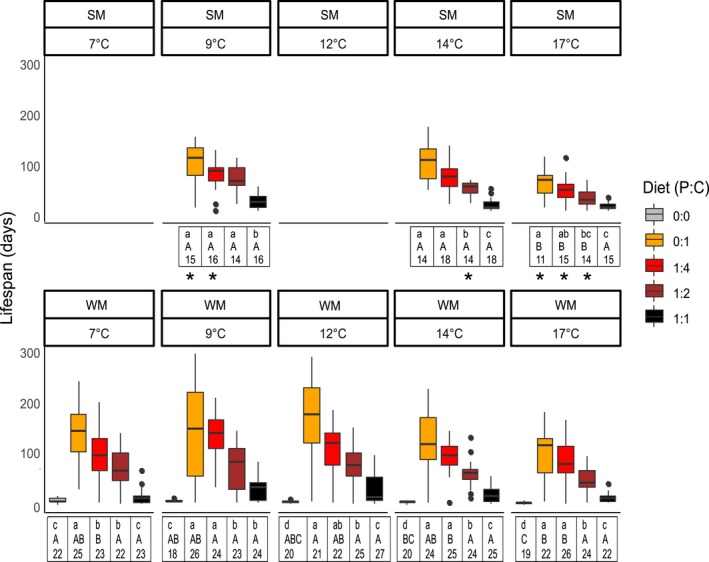
*Drosophila suzukii* WM and SM lifespan in variable diets and temperatures. The box represents the interquartile range, the line in the middle is the median, and whiskers represent extreme values within 1.5 times the interquartile range. Different lowercase letters represent differences between diets for each temperature (separately for WM and SM); different uppercase letters represent differences between temperatures for each diet (separately for WM and SM). The number represents sample size for each treatment. Asterisks represent diet and temperature treatments where SM and WM were significantly different

**Figure 2:**
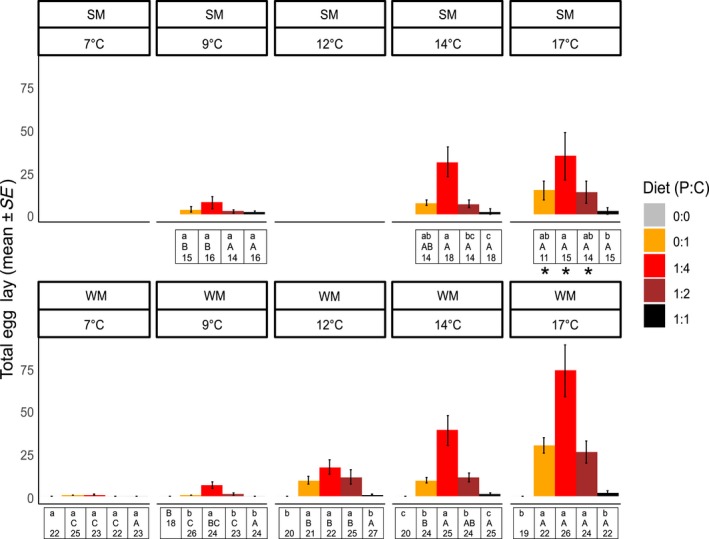
*Drosophila suzukii* WM and SM fecundity in variable diets and temperatures (total egg lay, mean ± *SE*). Different lowercase letters represent differences between diets for each temperature (separately for WM and SM); different uppercase letters represent differences between temperatures for each diet (separately for WM and SM). The number represents sample size for each treatment. Asterisks represent diet and temperature treatments where SM and WM were significantly different

**Figure 3:**
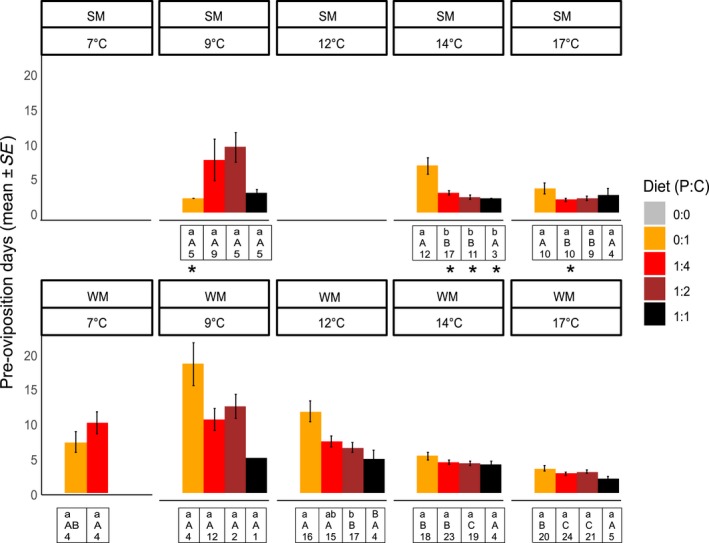
*Drosophila suzukii* WM and SM pre‐oviposition period in variable diets and temperatures (calendar days, mean ± *SE*). Different lowercase letters represent differences between diets for each temperature (separately for WM and SM); different uppercase letters represent differences between temperatures for each diet (separately for WM and SM). The number represents sample size for each treatment. Asterisks represent diet and temperature treatments where SM and WM were significantly different

WM pairs in rearing bottles were exposed to all diet treatments (P:C 0:0, 0:1, 1:4, 1:2, 1:1) and temperature regimes (7, 9, 12, 14, and 17°C). SM pairs were exposed to four diet treatments (P:C 0:1, 1:4, 1:2, 1:1) and three temperature regimes (9, 14 and 17°C). Preliminary observations showed that SM did not survive more than 2 days in P:C 0:0 diet; therefore, this diet was not tested in these trials. The lowest temperature was selected because in a previous study *D. suzukii* SM females did not lay eggs below 10°C (Tochen et al., 2014); hence, we excluded 7°C for SM. Sample sizes for each treatment are shown in Figures 1, 2, 3.

### 2.3 Experiment #1: Lifespan and oviposition in variable diets and temperatures

Overwintered WM females were 78 days old (since adult emergence, after cold hardening and overwintering) and SM were 1 day old (since emergence) when they were paired with males and placed into diet/temperature treatments. Previous studies that describe physiological differences between WM and SM (Kirkpatrick et al., 2018; Wong, Wallingford, Loeb, & Lee, 2018) have not taken into account the effect of an overwintering period. Therefore, rather than exposing WM and SM to the exact same pre‐treatment conditions, our intention was to simulate what WM would encounter with increasing temperatures after overwintering as adults, and what SM emerging in late summer would experience with decreasing temperatures during autumn.

Each week, we counted eggs present in the agar diet and replaced with fresh diet. Dead males were replaced with another sexually mature 3‐ to 4‐week‐old WM, or a 1‐ to 3‐day‐old SM, such that females were always paired with males for the duration of the experiment. Experiments were terminated when the female died. To evaluate differences in lifespan and pre‐oviposition period (time span before first egg laid), we used calendar days starting from the day when flies were placed in the diet/temperature treatments.

A nonparametric Kaplan–Meier analysis of survival with post hoc log‐rank tests with a Bonferroni correction (*α* = 0.05/number of pairwise comparisons) was performed to test for differences in median fly lifespan (LT50, in calendar days) among diets and temperatures. These analyses were carried out separately for WM and SM flies while comparing temperatures within a given diet or diets at a given temperature. To test the effects of temperature, diet, or fly morph on (a) total number of eggs per female (fecundity) and (b) pre‐oviposition period (in calendar days), we compared several general linear mixed models (GLMM) against a full model which included a temperature*diet*morph interaction as fixed effects and number of males as a random effect. The best fit was selected using the lowest Akaike's criterion information (AIC) and compared to the full model using an analysis of variance (ANOVA). To meet assumptions of normality and heteroscedasticity, variables were transformed using a Box–Cox lambda transformation. Post hoc HSD Tukey comparisons were then made comparing temperatures within a given diet, or diets at a given temperature for each morph. All data analyses henceforth were performed in RStudio (R Team, 2017); data were organized using the package “dplyr” (Wickham & Francois, 2016), the transformations and mixed linear model analyses were performed using the package “MASS” (Venables & Ripley, 2002) and “lme4” (Bates, Machler, Bolker, & Walker, 2015). Post hoc comparison groupings were done using the package “agricolae” (Mendiburu, 2017). All graphs were produced using the package “ggplot2” (Wickham, 2016).

### 2.4 Experiment #2: Nutrient profiles in different P:C diets

To determine how different diets affected nutrient body content, we measured macronutrient body contents (sugar, glycogen, lipids, and protein) in relation to P:C dietary ratio in SM and WM females. WM and SM flies were reared from the colonies maintained at USDA, under the same conditions described above. Cohorts of newly emerged WM and SM flies (from cornmeal diet) were placed in bottles in groups of 5–15 of mixed sexes and offered P:C 0:0, 0:1, 1:4, 1:2 and 1:1 agar diets during 7 days at 17°C (2 days for flies in 0:0 diet; *n* = 40 for each diet/morph treatment). For this experiment, we aimed to detect innate differences between WM and SM in body nutrient content; therefore, WM were not exposed to a simulated overwintering period and were exposed to diet treatments after emergence. After the diet exposure period, flies were frozen at −80°C and preserved for whole‐body nutrient content assays.

The contents of sugar, glycogen, and lipids on female flies were determined using a hot anthrone and vanillin assay, following a previous protocol (Tochen et al., 2016) adapted for 96‐well microplates (Wong, Cave et al., 2018). Protein content was measured using the Bradford assay (Jones, Hare, & Compton, 1989), previously adapted for *Drosophila* (Schmidt, Sebastian, Wilder, & Rypstra, 2012). A calibration standard was made by performing a Bradford assay on concentrations of 73, 80.3, 87.6, 94.9, 102.2, and 109.5 µg/ml of bovine gamma globulin (1.46 mg/ml; #500‐001 Bio‐Rad, Hercules CA, USA).

A general linear model (GLM) was performed using diet (P:C ratio) as a fixed effect and nutrient content as an outcome variable. This was done individually for each nutrient (protein, sugar, glycogen, and lipids) and each fly morph. Box–Cox or square root transformations were used as necessary to meet assumptions of normality and heteroscedasticity. Post hoc HSD Tukey and *t* tests were done on transformed data to determine differences between diets and fly morphs.

### 2.5 Experiment #3: Body nutrient depletion during overwintering

This experiment aimed to describe depletion of protein, lipids, sugar, and glycogen body content during simulated overwintering with no food resources. The flies for this experiment were WM *D. suzukii* females from a colony maintained at Cornell University, Geneva, NY (described previously in Wallingford et al., 2016). WM were reared and cold‐hardened under conditions as described above (see *Fly rearing*). Cohorts of 20 cold‐hardened WM (50:50 male:female) were transferred to vials (25 × 95 mm) with cellulose acetate stoppers (VWR International, Radnor PA, USA) containing 10 ml of water agar (10 g agar/L distilled water) and held at winter conditions (1°C, 12:12 hr L:D) for 5 weeks. Cohorts were removed from winter conditions weekly, and surviving females were preserved for whole‐body nutrient content analysis by freezing at −80°C. Assays to test for protein, sugar, lipids, and glycogen contents were performed as described above. To determine if body nutrient contents decreased during overwintering, we ran a linear model with week of overwintering as a continuous fixed effect, and nutrient content as an outcome variable, individually for protein, sugar, glycogen, and lipids.

## 3 RESULTS

### 3.1 Lifespan and oviposition in variable diets and temperatures

An average of 28.48% of WM females survived 5 weeks of overwintering at 1˚C, and these flies were subsequently placed in diet/temperature treatments. The lifespan of WM flies was significantly affected by diet and temperature (Supporting Information Table S1). WM flies receiving 0:1 diets lived longer than flies on 1:4 diets at 7°C, but had a similar lifespan at all other trialed temperatures. Flies on 0:1 diet lived longer compared to flies receiving 1:2 diet at all temperatures. Flies receiving 0:0 diet (starved) and flies receiving 1:1 diets had the shortest lifespan across all temperatures. Flies generally lived for longer periods at intermediate temperatures (9 and 12°C), except on 1:2 diet, where temperature did not have a significant effect on lifespan (Figure 1). The longest lifespan recorded for WM was 301 days (0:1, 9°C).

The lifespan of SM flies was significantly affected by diet and temperature, with the exception of 1:1 diet (Supporting Information Table S1). In SM, flies receiving 0:1 diet had a similar lifespan to flies on 1:4 diets in all temperatures and had a longer lifespan compared to flies on 1:2 diet at 14°C and 17°C. Flies receiving 1:1 diets had the shortest lifespan across all temperatures. Flies displayed a shorter lifespan at 17°C (the highest temperature) compared to 14 and 9°C; however, this effect was not observed when flies received 1:1 diet (Figure 1). The longest lifespan recorded for SM was 175 days (0:1, 14°C).

WM flies lived significantly longer than SM when receiving 0:1 and 1:4 diet at 9°C and 17°C. There were no differences in lifespan between morphs in flies fed 1:1 diet (Supporting Information Table S1, Figure 1).

The interactions between temperature, diet, and morph best explained fecundity (total eggs), and pre‐oviposition period in *D. suzukii* (Table 1, Supporting Information Tables S1 and S2). The fecundity of WM flies was significantly affected by diet and temperature (Supporting Information Table S1).WM flies receiving 1:4 diet had higher fecundity compared to 1:1 diets at all temperatures except 7°C. At this temperature, fecundity was very low, and there was oviposition only when flies were exposed to 0:1 and 1:4 diets (only four flies laid eggs in each of these diet treatments). Flies had higher fecundity at 17°C compared to 7, 9, and 12°C in all diets except 1:1. Oviposition in flies receiving 1:1 diet was generally low, although an analysis of variance suggested an effect of temperatures (Supporting Information Table S1), a post hoc with adjusted values for multiple comparisons did not detect differences among temperatures (Figure 2). The highest number of eggs laid recorded for WM was 352 eggs (1:4, 17°C).

**Table 1:** Akaike's information criterion (AIC) and comparison with full model for multiple models including temperature, diet, and fly morph as fixed effects, and number of males as random effects

Outcome variable = fecundity	AIC	ANOVA parameters compared to full model		
		*χ* ^2^	*df*	*p*
Terms included in model				
Temperature*Diet*Morph	1,256.5			
Temperature + Diet + Morph	1,341.5	138.99	27	<0.01
Temperature*Diet	1,258.4	25.95	12	0.01
Temperature + Diet	1,339.8	139.33	28	<0.01
Temperature*Morph	1,596.3	397.84	29	<0.01
Temperature + Morph	1,598.9	404.45	31	<0.01
Diet*Morph	1,534.8	334.32	28	<0.01
Diet + Morph	1,529.7	335.24	31	<0.01
Temperature	1,602.6	410.09	31	<0.01
Diet	1,533.6	341.16	32	<0.01
Morph	1,734.5	548.07	35	<0.01
None (null model)	1,751	566.49	36	<0.01
Outcome variable = pre‐oviposition period				
Temperature*Diet*Morph	60.8			
Temperature + Diet + Morph	66.9	48.09	21	<0.01
Temperature*Diet	101	64.26	12	0.01
Temperature + Diet	99.5	82.69	22	<0.01
Temperature*Morph	84.5	67.7	22	<0.01
Temperature + Morph	82.6	69.77	24	<0.01
Diet*Morph	157.7	140.96	22	<0.01
Diet + Morph	153.4	142.62	25	<0.01
Temperature	115.3	104.52	25	<0.01
Diet	187.1	178.2	26	<0.01
Morph	162.5	157.7	28	<0.01
None (null model)	197.6	194.83	29	<0.01

The fecundity of SM flies was significantly affected by diet and temperature (Supporting Information Table S1). SM flies receiving 1:4 diet had higher fecundity than flies receiving 1:1 diet within all temperatures except 9°C, where fecundity was similar on all diets. Fecundity was higher at 17°C compared to 9°C in flies receiving 0:1 and 1:4 diets. Oviposition levels of flies receiving 1:1 and 1:2 diets were generally low and did not vary significantly between temperatures (Figure 2). The highest number of eggs laid recorded for SM was 183 eggs (1:4, 17°C).

WM receiving 0:1, 1:4, and 1:2 diet laid more eggs than SM only in 17°C. There were no differences in fecundity between morphs in other temperatures or in 1:1 diet (Supporting Information Table S1, Figure 2).

The pre‐oviposition period in WM flies was significantly affected by both diet and temperature (Supporting Information Table S1). WM females receiving 0:1 diet had a significantly longer pre‐oviposition period compared to females receiving the other diets only at 12°C (11 weeks), while the pre‐oviposition periods were not different between diets at all other temperatures. WM females had shorter pre‐oviposition periods at 17°C compared to 9°C, and 12°C in all diets. For flies on 1:1 diet, although an analysis of variance suggested an effect of temperatures (Supporting Information Table S1), a post hoc with adjusted values for multiple comparisons did not detect differences among temperatures (Figure 3).

The pre‐oviposition period in SM flies was also significantly affected by diet and temperature (Supporting Information Table S1). At 14°C, SM females receiving 0:1 diet had the longest pre‐oviposition period compared to females receiving all other diet treatments, but there was no diet effect at 9°C and 17°C (Figure 3). SM females receiving 1:4 and 1:2 diet had a longer pre‐oviposition period at 9°C compared to the other temperatures, but females receiving 0:1 and 1:1 diet did not have different pre‐oviposition periods among temperatures (Figure 3).

SM flies receiving 0:1 had a shorter pre‐oviposition period than WM at 9°C, but the sample size in this temperature was small, and there were no differences in other temperatures. SM receiving 1:4 diet had shorter pre‐oviposition periods than SM at 14°C and 17°C (Supporting Information Table S1, Figure 3).

### 3.2 Nutrient profiles and depletion

**Figure 4:**
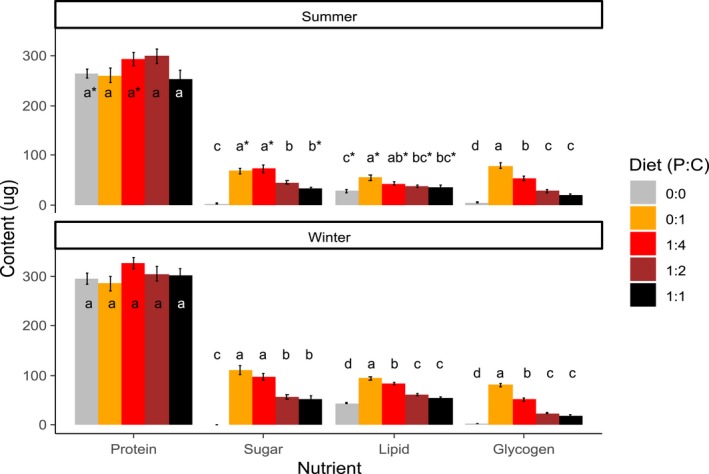
*Drosophila suzukii* winter (WM) and summer morph (SM) total body content of protein, sugar, lipid, and glycogen exposed to different diets (Protein:Carbohydrate 0:0, 0:1, 1:4, 1:2, 1:1; mean ± *SE*) for seven days at 17°C. Bars with same letters within each nutrient are not significantly different (Tukey HSD). Asterisks represent significant differences between WM and SM within each diet (*t* test).

GLM parameters to test for differences in body nutrient content are summarized in Table 2. In all diets, WM had higher lipid and similar glycogen contents than SM. WM flies receiving 0:1 and 1:2 diets had similar protein and sugar contents as SM; in all other diets, WM had higher protein and sugar contents than SM (Figure 4).

**Table 2:** General linear model parameters on transformed values for the effect of diet and temperature on nutrient content in WM and SM flies

Protein	Winter morphs			Summer morphs		
	*R* ^2^ < 0.01, *df* = 4, 195, *F* = 0.98, *p* = 0.41			*R* ^2^ = 0.01, *df* = 4, 195, *F* = 1.97, *p* = 0.09		
	*β*	*t*	*p*	*β*	*t*	*p*
Intercept	16.60	36.20	<0.01	15.76	29.80	<0.01
Diet (0:0)	0.44	0.68	0.49	0.37	0.50	0.61
Diet (1:4)	1.26	1.95	0.05	1.07	1.44	0.15
Diet (1:2)	0.55	0.86	0.39	1.27	1.70	0.09
Diet (1:1)	0.55	0.85	0.39	−0.51	−0.69	0.49

Sugar	*R* ^2^ = 0.59, *df* = 4, 195, *F* = 74.17, *p* < 0.01			*R* ^2^ = 0.61, *df* = 4, 195, *F* = 79.94, *p* < 0.01		
	*β*	*t*	*p*	*β*	*t*	*p*
Intercept	10.09	24.31	<0.01	8.06	25.23	<0.01
Diet (0:0)	−9.05	−15.42	<0.01	−6.86	−15.18	<0.01
Diet (1:4)	−0.55	−0.94	0.34	0.06	0.14	0.88
Diet (1:2)	−3.01	−5.13	<0.01	−1.60	−3.58	<0.01
Diet (1:1)	−3.70	−6.31	<0.01	−2.85	−6.36	<0.01

Lipid	*R* ^2^ = 0.56, *df* = 4, 195, *F* = 65.11, *p* < 0.01			*R* ^2^ = 0.10, *df* = 4, 195, *F* = 7.06, *p* < 0.01		
	*β*	*t*	*p*	*β*	*t*	*p*
Intercept	9.68	61.82	<0.01	7.09	23.66	<0.01
Diet (0:0)	−3.05	−13.78	<0.01	−2.15	−5.03	<0.01
Diet (1:4)	−0.59	−2.66	<0.01	−0.80	−1.89	0.06
Diet (1:2)	−1.89	−8.56	<0.01	−1.18	−2.78	<0.01
Diet (1:1)	−2.38	−10.75	<0.01	−1.46	−3.45	<0.01

Glycogen	*R* ^2^ = 0.79, *df* = 4, 195, *F* = 195.5, *p* < 0.01			*R* ^2^ = 0.64, *df* = 4, 195, *F* = 91.41, *p* < 0.01		
	*β*	*t*	*p*	*β*	*t*	*p*
Intercept	8.88	44.23	<0.01	8.58	31.62	<0.01
Diet (0:0)	−7.29	−25.68	<0.01	−6.75	−17.59	<0.01
Diet (1:4)	−1.78	−6.27	<0.01	−1.63	−4.26	<0.01
Diet (1:2)	−4.13	−14.56	<0.01	−3.54	−9.23	<0.01
Diet (1:1)	−4.75	−16.73	<0.01	−4.44	−11.56	<0.01

Note

Regression values are compared to a baseline P:C 0:1 diet.

In WM and SM, body sugar content was higher in flies receiving 0:1 and 1:4 diets compared to the other diets. Body glycogen content was highest in flies receiving 0:1 diet and lowest in 1:1 and 0:0 diet in both morphs. The main difference between SM and WM was that SM flies had similar lipid contents on 0:1 and 1:4 diets, higher than on 1:2, 1:1 and 0:0 diets, whereas WM flies had higher lipid contents on 0:1 compared to 1:4 diet, and on 1:2 and 1:1 compared to 0:0 diet. Protein content was, however, not affected by diet in either morph (Figure 4, Table 2).

**Figure 5:**
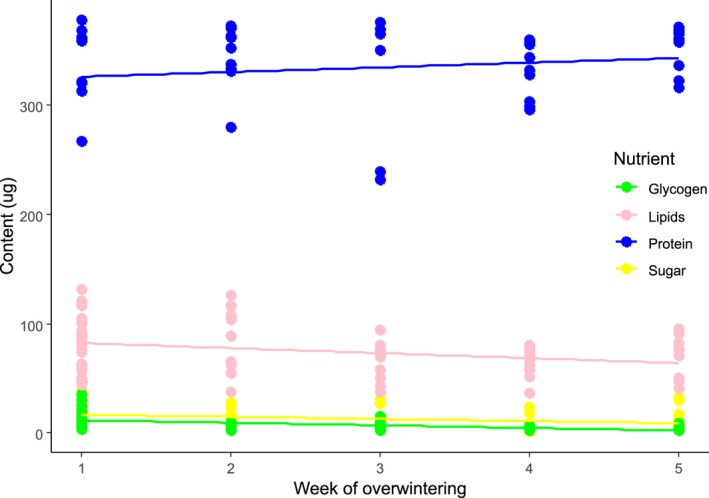
Weekly total body content of protein, sugar, lipid, and glycogen in *Drosophila suzukii* WM females during five weeks of overwintering at 1°C

The body protein content of overwintering WM females did not decrease as the overwintering period progressed (*R* ^2^ = 0.01, *df* = 1, 44, *F* = 0.52, *p* = 0.47), but there was, however, a significant decrease in sugar (*R* ^2^ = 0.09, *df* = 1, 70, *F* = 7.31, *p* < 0.01), glycogen (*R* ^2^ = 0.18, *df* = 1, 44, *F* = 16.19, *p* < 0.01), and lipid (*R* ^2^ = 0.08, *df* = 1, 68, *F* = 6.05, *p* = 0.01; Figure 5).

## 4 DISCUSSION

In general, at intermediate and higher temperatures, WM and SM flies had similar lifespans on 0:1 and 1:4 diets, while lifespan decreased in flies receiving high‐protein 1:1 diets. There was a trend for both WM and SM to have the highest fecundity on low‐protein (1:4) diets. This suggests that in these conditions there is little trade‐off between lifespan and fecundity, and *D. suzukii* overall benefits the most from low‐protein diets.

There were some key differences in lifespan and fecundity between WM and SM. For instance, WM on 0:1 and 1:4 diets had longer lifespans than SM (at 9 and 17°C). Furthermore, when exposed to more favorable conditions (1:4 diet, 17°C) WM also had higher fecundity than SM. This is remarkable, given that WM females had to recover from exposure to an extreme environmental stressor, as is overwintering at near‐freezing temperatures with no food sources. Body nutrient contents suggest that there are some differences in nutrient metabolism between SM and WM which may be linked to these differences in lifespan and fecundity. Specifically, WM flies receiving 1:4 diet had higher body protein, lipid, and sugar contents compared to SM, which may be linked to longer lifespan and higher fecundity. The main contrast between WM and SM nutrient contents in different diets was in lipid contents; we found that body lipid contents were significantly higher in WM flies receiving 0:1 compared to 1:4 diets, while lipid contents were similar between SM flies receiving 0:1 and 1:4 diets. This suggests that when offered carbohydrate‐only diets, WM flies are more efficient at converting sugars into lipids than SM are, potentially for long‐term energy storage, or post‐overwintering replenishment. This was expected, as insect stages that are destined for overwintering commonly have higher lipid reserves, essential for survival (Sinclair & Marshall, 2018). WM had little depletion of protein content during overwintering and can quickly replenish sugar and lipid content on a carbohydrate‐only diet to survive at low temperatures, meaning that WM *D. suzukii* has a great fecundity potential as temperatures increase during spring.

Because SM did not have to recover from an overwintering period, we expected that at lower temperatures SM flies would have increased fecundity with the addition of dietary protein. We found, however, that at 9°C (the lowest temperature tested for SM), female SM receiving 0:1 and 1:4 diet had similar fecundity, while WM females had higher fecundity on 1:4 diet compared to 0:1 diet. This suggests that at around 9°C, SM are not investing energy in reproduction, and therefore do not benefit from additional dietary protein, whereas WM can already benefit from low dietary protein for reproduction.

At 7°C, WM females survived a week or longer on 0:0 diet, meaning that remaining body nutrient reserves left at the end of the overwintering period can enable females to survive a short period and disperse while searching for optimal resources (Sinclair, 2015). At very low temperatures (7°C), post‐overwintering WM flies had longer lifespans on carbohydrate‐only 0:1 diets compared to the other diets, and the addition of dietary protein negatively affected lifespan. This suggests that, at very low temperatures, post‐overwintering WM benefit more from having a carbohydrate‐only diet. This is further evidenced by the fact that at 7°C, the addition of dietary protein did not enhance fecundity, or reduce pre‐oviposition periods. At very low temperatures, it is possible that WM flies do not invest many resources in reproduction, and instead are replenishing lipids, sugars, and glycogen lost during overwintering. Sugars are the main macronutrients used in the biosynthesis of glycogen and lipids; we indeed found that when exposed to 0:1 diet, WM female flies had higher contents of glycogen and sugars than flies exposed to higher protein diets.

At moderate temperature conditions, our results follow the general trend of low‐protein diets being optimal for *Drosophila* adult lifespan (Bruce et al., 2013). Some studies have, however, reported slightly different optimal dietary P:C ratios for *Drosophila* lifespan; for instance, it has been shown that *D. melanogaster* has a longer lifespan in low‐protein diets compared to carbohydrate‐only (0:1) diets (Jensen et al., 2015; Lee, 2015; Lee et al., 2008). Additionally, *D. melanogaster* fed 1:4 diet had longer lifespan compared to 1:16 diet (Lee & Jang, 2014). The fact that *D. suzukii* receiving low‐protein diets did not live longer than flies in a carbohydrate‐only diet (as opposed to *D. melanogaster)* might relate to the gut microbiota and yeast microbes associated with *D. suzukii*. These microbiota might enable this species to survive and flourish in very low‐protein and carbohydrate‐only environments as is typically found in ripe fruit (Bing, Gerlach, Loeb, & Buchon, 2018; Hamby & Becher, 2016), compared to closely related *Drosophila* species. Unlike *D. melanogaster, D. suzukii* has evolved to develop in ripening fruit (Karageorgi et al., 2017), which presumably has lower levels of protein associated with fungi compared to rotting fruit. As a consequence, this life trait can make a difference in the dietary P:C levels that are optimal for *D. suzukii* adult fitness compared to closely related Drosophilids (Jaramillo et al., 2015; Silva‐Soares et al., 2017).

Other studies also found that *D. suzukii* fed protein + sugar diets as adults matured eggs, whereas those fed only sugar diets had very few to no eggs (Plantamp et al., 2017; Wong, Wallingford et al., 2018). Similarly, one study showed that *D. melanogaster* has the highest egg production on P:C 1:4 diet (Lee et al., 2008), but others have reported that *D. melanogaster* has higher fecundity in medium‐ (1:2) or high‐protein (4:1) diets compared to low‐protein (1:4) diets (Jensen et al., 2015; Lee, 2015). While differences in fecundity may be in part attributed to different diets among studies, gut microbiota might also impact this parameter. For instance, *D. melanogaster* flies infected with the gut bacteria *Wolbachia* display maximum reproductive rates when exposed to P:C 1:1 diets, as opposed to flies not infested with *Wolbachia,* which displayed a maximum reproductive rate when exposed to P:C 1:2 diets (Ponton et al., 2015). *Wolbachia* infection in *D. suzukii* can vary between 20% and 70% (Tochen et al., 2014), and because the incidence of *Wolbachia* or other potentially important microbes (Chandler, James, Jospin, & Lang, 2014; Hamby & Becher, 2016) is not commonly tested in *Drosophila* studies, this variability may explain how different studies report different optimal P:C diets for fecundity and lifespan in *Drosophila* species. Dietary P:C can affect fertility in flies as well (% larvae hatch; Oviedo et al., 2011), so future studies should address comparisons and potential trade‐offs between fecundity and fertility.

This study showed that *D. suzukii* WM and SM had a shorter lifespan on high‐protein diets compared to low‐protein or carbohydrate‐only diets, which is consistent with results from previous studies in *Drosophila* (Jensen et al., 2015; Lee, 2015; Ponton et al., 2015). To better understand why increased levels of dietary protein negatively impact *D. suzukii* lifespan and fecundity, we looked at the whole‐body nutrient contents in flies receiving the different diets. Flies exposed to 1:1 diets had similar body protein content compared to flies exposed to 0:1 diet. The similar body protein content suggests that flies incorporate dietary protein up to a threshold and above this threshold; they are possibly spending additional energy excreting excess protein (Grandison, Piper, & Partridge, 2009). This increased cost may negatively affect both fecundity and lifespan. Organisms with imbalanced diets relative to their metabolic needs can die earlier or produce fewer offspring (Simpson & Raubenheimer, 2011), or be less well suited to withstand adverse conditions. For instance, *D. melanogaster* flies receiving high‐protein diets have lower starvation resistance (Lee & Jang, 2014) and lower lipid reserves (Ponton et al., 2015) than flies fed lower protein diets. It is also possible that flies receiving suboptimal high‐protein diets feed less frequently, and thus are dying of starvation. This explanation is, however, unlikely, as it has been shown that *Drosophila* exposed to no‐choice high‐protein liquid diets still consume high quantities of diet (Fanson et al., 2012). We consistently found that both WM and SM individuals receiving 1:1 diets had lower sugar, lipid, and glycogen body contents than flies receiving 0:1 or 1:4 diets. This supports the idea that flies receiving suboptimal high‐protein diets sacrifice essential nutrients that could have been used either to increase survival and/or energy storage. In this study, we only manipulated dietary protein and carbohydrate content, but the effect of fluctuating dietary lipid and specific amino‐acids contents should also be examined in future studies.

We expected that the addition of dietary protein would accelerate oogenesis in WM and SM, resulting in shorter pre‐oviposition periods. There was not a clear trend to support this, as WM and SM flies exposed to carbohydrate‐only 0:1 diet had significantly longer pre‐oviposition periods compared to flies receiving dietary protein only at intermediate temperatures (12°C for WM and 14°C for SM). This suggests that at higher temperatures, females will begin oogenesis regardless of macronutrient intake. At 7°C, WM could, however, only resume oviposition in 0:1 and 1:4 diets. As expected, pre‐oviposition periods did tend to decrease with increasing temperatures in WM and SM females exposed to 1:4 and 1:2 diets. It has previously been reported that 100% of female *D. suzukii* reared at 15°C for 20 days contained mature eggs, while only 20% of females reared at 11°C had mature eggs (Toxopeus et al., 2016). Likewise, female *D. suzukii* from outdoor field conditions have more mature abdominal eggs with increasing degree‐day accumulation and above 10°C (Grassi et al., 2018). SM could lay eggs below 10°C, and WM laid eggs as cold as 7°C, lower temperatures than those previously reported (Ryan et al., 2016; Tochen et al., 2014). These results provide an insight about the dietary resources needed by post‐overwintering WM females in the field, suggesting that WM flies cannot mature eggs at very low temperatures when feeding on high‐protein diets.

Most studies on *D. suzukii* adult nutrition have focused on general food sources (Jaramillo et al., 2015; Plantamp et al., 2017; Stockton et al., 2018; Tochen et al., 2016). Although all these studies provide insight on the links between diet composition and *D. suzukii* fitness in different food sources, they do not address the effect of specific dietary macronutrients on adult *D. suzukii* survival and fecundity. To date, two other studies have examined the nutritional framework of SM *D. suzukii* in relation to its dietary P:C intake at the larval stage at a single temperature, while this study examined both SM and WM adult intake at a range of temperatures. One study showed that larval survival was highest on P:C 1:2 diets, while survival was decreased at lower protein diets (1:16, no carbohydrate‐only diet was tested; Silva‐Soares et al., 2017). Similarly, another study showed that more *D. suzukii* larvae survive on protein‐rich diets (P:C 24:1), while survival was decreased at lower protein diets (P:C 1:12; Young, Buckiewicz, & Long, 2018). Comparing with these studies, our study suggests that different developmental stages may have different dietary requirements in *D. suzukii*. In contrast with larval experiments, our work showed increased adult survival at low‐protein or carbohydrate‐only diets, while high‐protein diets were detrimental for adult survival. Protein intake is likely more important during the larval stage, as larvae require protein to build tissue as they grow in size and metamorphose. In contrast, adult flies may require lower levels of protein for egg production but not for growth, and any extra dietary protein involves spending additional energy for excretion.

As we strive to understand the expansion potential of invasive organisms, it is important to understand the role that abiotic factors play on their fitness. Phenotypic plasticity undoubtedly conveys an adaptive benefit, as WM can successfully survive and reproduce in challenging temperature conditions where SM would be disadvantaged. The results of this study can help elucidate the dietary trade‐offs that WM and SM insects make before and after the dormant period, as temperature and food resources fluctuate. There is evidence that Drosophilid and Tephritid flies regulate their nutritional intake to reach optimal P:C dietary ratios (Fanson et al., 2012; Oviedo et al., 2011); this means that throughout the year, WM and SM must find the most adequate nutrient composition to increase its fitness. This might be more challenging during and right after winter, when protein sources are still abundant (i.e., fungi and fecal matter), but some sugar sources (i.e., floral blooms, honeydew, ripe, and overripe fruit) can be scarcer. As spring temperatures increase and trigger oogenesis, the lifespan and high fecundity potential of WM females depend on finding appropriate carbohydrate sources to balance protein intake. While SM can more easily find multiple food sources to balance their carbohydrate and protein intake for a longer lifespan and egg production, their fecundity will likely decrease with lowering temperatures during autumn even with optimal diet. This study provides valuable insights on the success of polyphagous invasive insect adaptation, and the dietary requirements for the successful persistence of populations during suboptimal temperature and variable dietary conditions.

## CONFLICT OF INTEREST

The authors declare no competing interests.

## AUTHOR CONTRIBUTIONS

JL, AW, GL, VW, and DR designed the experiments. DR, GT, JB, and ILS performed the diet experiments and collected the data; AW maintained the overwintering colonies for nutrient assays. DR and VW analyzed the data. DR wrote the manuscript; all authors contributed to manuscript editing and approved of the final version.

## ETHICS APPROVAL

This study does not involve human participants or use of vertebrates. The use of insects does not require ethics approval.

## Data Availability

All the data used in this study is accessible in Dryad data repository https://doi.org/10.5061/dryad.84jr187.

## References

[ece34928-bib-0001] Allen, M. J. (2007). What makes a fly enter diapause? Fly, 1, 307–310. 10.4161/fly.5532 18820432

[ece34928-bib-0002] Bates, D. , Machler, M. , Bolker, B. M. , & Walker, S. C. (2015). Fitting Linear Mixed‐Effects Models using lme4. Journal of Statistical Software, 67, 1–48.

[ece34928-bib-0003] Beukeboom, L. W. (2018). What makes an insect invasive? An introduction. Entomologia Experimentalis Et Applicata, 166, 149–150. 10.1111/eea.12667

[ece34928-bib-0004] Bing, X. L. , Gerlach, J. , Loeb, G. , & Buchon, N. (2018). Nutrient‐dependent impact of microbes on *Drosophila suzukii* development. Mbio, 9, e02199‐17 10.1128/mBio.02199-17 29559576 PMC5874910

[ece34928-bib-0005] Bruce, K. D. , Hoxha, S. , Carvalho, G. B. , Yamada, R. , Wang, H. D. , Karayan, P. , … Ja, W. W. (2013). High carbohydrate‐low protein consumption maximizes *Drosophila* lifespan. Experimental Gerontology, 48, 1129–1135. 10.1016/j.exger.2013.02.003 23403040 PMC3687007

[ece34928-bib-0006] Burggren, W. W. (2017). Epigenetics in insects: mechanisms, phenotypes and ecological and evolutionary implications, Vol. 53 In Verlinden H. (Ed.), Insect epigenetics (pp. 1–30). New York, NY: Elsevier.

[ece34928-bib-0007] Chandler, J. A. , James, P. M. , Jospin, G. , & Lang, J. M. (2014). The bacterial communities of *Drosophila suzukii* collected from undamaged cherries. Peerj, 2, e474 10.7717/peerj.474 25101226 PMC4121540

[ece34928-bib-0008] Chen, Y. G. , Ciaramitaro, T. , & Poland, T. M. (2011). Moisture content and nutrition as selection forces for emerald ash borer larval feeding behaviour. Ecological Entomology, 36, 344–354. 10.1111/j.1365-2311.2011.01278

[ece34928-bib-0009] Conti, B. (2008). Considerations on temperature, longevity and aging. Cellular and Molecular Life Sciences, 65, 1626–1630. 10.1007/s00018-008-7536-1 18425417 PMC2574693

[ece34928-bib-0010] Dalton, D. T. , Walton, V. M. , Shearer, P. W. , Walsh, D. B. , Caprile, J. , & Isaacs, R. (2011). Laboratory survival of *Drosophila suzukii* under simulated winter conditions of the Pacific Northwest and seasonal field trapping in five primary regions of small and stone fruit production in the United States. Pest Management Science, 67, 1368–1374. 10.1002/ps.2280 22021034

[ece34928-bib-0011] Dos Santos, L. A. , Mendes, M. F. , Kruger, A. P. , Blauth, M. L. , Gottschalk, M. S. , & Garcia, F. R. (2017). Global potential distribution of *Drosophila suzukii* (Diptera, Drosophilidae). PLoS ONE, 12, e0174318 10.1371/journal.pone.0174318 28323903 PMC5360346

[ece34928-bib-0012] Fanson, B. G. , & Taylor, P. W. (2012). Protein:carbohydrate ratios explain life span patterns found in Queensland fruit fly on diets varying in yeast:sugar ratios. Age, 34, 1361–1368. 10.1007/s11357-011-9308-3 21904823 PMC3528373

[ece34928-bib-0013] Fanson, B. G. , Yap, S. , & Taylor, P. W. (2012). Geometry of compensatory feeding and water consumption in *Drosophila melanogaster* . Journal of Experimental Biology, 215, 766–773. 10.1242/jeb.066860 22323199

[ece34928-bib-0014] Fordyce, J. A. (2006). The evolutionary consequences of ecological interactions mediated through phenotypic plasticity. Journal of Experimental Biology, 209, 2377–2383. 10.1242/jeb.02271 16731814

[ece34928-bib-0015] Fraimout, A. , Jacquemart, P. , Villarroel, B. , Aponte, D. J. , Decamps, T. , Herrel, A. , … Debat, V. (2018). Phenotypic plasticity of *Drosophila suzukii* wing to developmental temperature: Implications for flight. Journal of Experimental Biology, 221 10.1242/jeb.166868 29987053

[ece34928-bib-0016] Garnas, J. R. , Auger‐Rozenberg, M. A. , Roques, A. , Bertelsmeier, C. , Wingfield, M. J. , Saccaggi, D. L. , … Slippers, B. (2016). Complex patterns of global spread in invasive insects: Eco‐evolutionary and management consequences. Biological Invasions, 18, 935–952. 10.1007/s10530-016-1082-9

[ece34928-bib-0017] Grandison, R. C. , Piper, M. D. W. , & Partridge, L. (2009). Amino‐acid imbalance explains extension of lifespan by dietary restriction in *Drosophila* . Nature, 462, 1061–1064. 10.1038/nature08619 19956092 PMC2798000

[ece34928-bib-0018] Grassi, A. , Gottardello, A. , Dalton, D. T. , Tait, G. , Rendon, D. , Ioriatti, C. , … Walton, V. M. (2018). Seasonal reproductive biology of *Drosophila suzukii* (Diptera: Drosophilidae) in temperate climates. Environmental Entomology, 47, 166–174. 10.1093/ee/nvx195 29281089

[ece34928-bib-0019] Hamby, K. A. , & Becher, P. G. (2016). Current knowledge of interactions between *Drosophila suzukii* and microbes, and their potential utility for pest management. Journal of Pest Science, 89, 621–630. 10.1007/s10340-016-0768-1

[ece34928-bib-0020] Irwin, M. T. , Raharison, J.‐L. , Raubenheimer, D. R. , Chapman, C. A. , & Rothman, J. M. (2015). The nutritional geometry of resource scarcity: Effects of lean seasons and habitat disturbance on nutrient intakes and balancing in wild sifakas. PLoS ONE, 10(6), e0128046 10.1371/journal.pone.0128046 26061401 PMC4464895

[ece34928-bib-0021] Jaramillo, S. L. , Mehlferber, E. , & Moore, P. J. (2015). Life‐history trade‐offs under different larval diets in *Drosophila suzukii* (Diptera: Drosophilidae). Physiological Entomology, 40, 2–9. 10.1111/phen.12082

[ece34928-bib-0022] Jensen, K. , McClure, C. , Priest, N. K. , & Hunt, J. (2015). Sex‐specific effects of protein and carbohydrate intake on reproduction but not lifespan in *Drosophila melanogaster* . Aging Cell, 14, 605–615. 10.1111/acel.12333 25808180 PMC4531074

[ece34928-bib-0023] Jones, C. G. , Hare, J. D. , & Compton, S. J. (1989). Measuring plant protein with the bradford assay.1. Evaluation and standard method. Journal of Chemical Ecology, 15, 979–992. 10.1007/bf01015193 24271900

[ece34928-bib-0024] Karageorgi, M. , Braecker, L. B. , Lebreton, S. , Minervino, C. , Cavey, M. , Siju, K. P. , … Prudhomme, B. (2017). Evolution of multiple sensory systems drives novel egg‐laying behavior in the fruit pest *Drosophila suzukii* . Current Biology, 27, 847–853. 10.1016/j.cub.2017.01.055 28285999 PMC5364372

[ece34928-bib-0025] Kay, A. D. , Zumbusch, T. , Heinen, J. L. , Marsh, T. C. , & Holway, D. A. (2010). Nutrition and interference competition have interactive effects on the behavior and performance of Argentine ants. Ecology, 91, 57–64. 10.1890/09-0908.1 20380196

[ece34928-bib-0026] Kenis, M. , Tonina, L. , Eschen, R. , Van der Sluis, B. , Sancassani, M. , Mori, N. , … Helsen, H. (2016). Non‐crop plants used as hosts by *Drosophila suzukii* in Europe. Journal of Pest Science, 89, 735–748. 10.1007/s10340-016-0755-6 28275324 PMC5318492

[ece34928-bib-0027] Kirkpatrick, D. M. , Leach, H. L. , Xu, P. , Dong, K. , Isaacs, R. , & Gut, L. J. (2018). Comparative antennal and behavioral responses of summer and winter morph *Drosophila suzukii* (Diptera: Drosophilidae) to ecologically relevant volatiles. Environmental Entomology, 47, 700–706. 10.1093/ee/nvy046 29668908

[ece34928-bib-0028] Landsberg, L. (2012). Core temperature: A forgotten variable in energy expenditure and obesity? Obesity Reviews, 13, 97–104. 10.1111/j.1467-789X.2012.01040.x 23107263

[ece34928-bib-0029] Le Couteur, D. G. , Solon‐Biet, S. , Cogger, V. C. , Mitchell, S. J. , Senior, A. , de Cabo, R. , … Simpson, S. J. (2016). The impact of low‐protein high‐carbohydrate diets on aging and lifespan. Cellular and Molecular Life Sciences, 73, 1237–1252. 10.1007/s00018-015-2120-y 26718486 PMC11108352

[ece34928-bib-0030] Leclaire, M. , & Brandl, R. (1994). Phenotypic plasticity and nutrition in a phytophagous insect – Consequences of colonizing a new host. Oecologia, 100, 379–385. 10.1007/bf00317858 28306925

[ece34928-bib-0031] Lee, J. C. , Bruck, D. J. , Dreves, A. J. , Ioriatti, C. , Vogt, H. , & Baufeld, P. (2011). In focus: Spotted wing drosophila, *Drosophila suzukii*, across perspectives. Pest Management Science, 67, 1349–1351. 10.1002/ps.2271 21990168

[ece34928-bib-0032] Lee, J. C. , Dreves, A. J. , Cave, A. M. , Kawai, S. , Isaacs, R. , Miller, J. C. , … Bruck, D. J. (2015). Infestation of wild and ornamental noncrop fruits by *Drosophila suzukii* (Diptera: Drosophilidae). Annals of the Entomological Society of America, 108, 117–129. 10.1093/aesa/sau014

[ece34928-bib-0033] Lee, K. P. (2015). Dietary protein:carbohydrate balance is a critical modulator of lifespan and reproduction in *Drosophila melanogaster:* A test using a chemically defined diet. Journal of Insect Physiology, 75, 12–19. 10.1016/j.jinsphys.2015.02.007 25728576

[ece34928-bib-0034] Lee, K. P. , & Jang, T. (2014). Exploring the nutritional basis of starvation resistance in *Drosophila melanogaster* . Functional Ecology, 28, 1144–1155. 10.1111/1365-2435.12247

[ece34928-bib-0035] Lee, K. P. , Simpson, S. J. , Clissold, F. J. , Brooks, R. , Ballard, J. W. , Taylor, P. W. , … Raubenheimer, D. (2008). Lifespan and reproduction in *Drosophila:* New insights from nutritional geometry. Proceedings of the National Academy of Sciences of the United States of America, 105, 2498–2503. 10.1073/pnas.0710787105 18268352 PMC2268165

[ece34928-bib-0036] Lehmann, P. , Van der Bijl, W. , Nylin, S. , Wheat, C. W. , & Gotthard, K. (2017). Timing of diapause termination in relation to variation in winter climate. Physiological Entomology, 42, 232–238. 10.1111/phen.12188

[ece34928-bib-0037] Mendiburu, F. (2017). agricolae: Statistical procedures for agricultural research. Retrieved from https://CRAN.R-project.org/package=agricolae

[ece34928-bib-0038] Moczek, A. P. (2010). Phenotypic plasticity and diversity in insects. Philosophical Transactions of the Royal Society B‐Biological Sciences, 365, 593–603. 10.1098/rstb.2009.0263 PMC2817146 20083635

[ece34928-bib-0039] Oviedo, A. , Nestel, D. , Papadopoulos, N. T. , Ruiz, M. J. , Prieto, S. C. , Wilnik, E. , & Vera, M. T. (2011). Management of protein intake in the fruit fly *Anastrepha fraterculus* . Journal of Insect Physiology, 57, 1622–1630. 10.1016/j.jinsphys.2011.08.013 21896276

[ece34928-bib-0040] Plantamp, C. , Estragnat, V. , Fellous, S. , Desouhant, E. , & Gibert, P. (2017). Where and what to feed? Differential effects on fecundity and longevity in the invasive *Drosophila suzukii* . Basic and Applied Ecology, 19, 56–66. 10.1016/j.baae.2016.10.005

[ece34928-bib-0041] Ponton, F. , Wilson, K. , Holmes, A. , Raubenheimer, D. , Robinson, K. L. , & Simpson, S. J. (2015). Macronutrients mediate the functional relationship between Drosophila and Wolbachia. Proceedings of the Royal Society B. Biological Sciences, 282, 20142029 10.1098/rspb.2014.2029 PMC4298205 25520356

[ece34928-bib-0042] R Team (2017). R: A language and environment for statistical computing. Vienna, Austria: R Foundation for Statistical Computing.

[ece34928-bib-0043] Rendon, D. , Buser, J. , Tait, G. , Lee, J. C. , & Walton, V. M. (2018). Survival and fecundity parameters of two *Drosophila suzukii* (Diptera: Drosophilidae) morphs on variable diet under suboptimal temperatures. Journal of Insect Science, 18(6). 10.1093/jisesa/iey113 PMC6237241 30445636

[ece34928-bib-0044] Rho, M. S. , & Lee, K. P. (2016). Balanced intake of protein and carbohydrate maximizes lifetime reproductive success in the mealworm beetle, *Tenebrio molitor* (Coleoptera: Tenebrionidae). Journal of Insect Physiology, 91, 93–99. 10.1016/j.jinsphys.2016.07.002 27405009

[ece34928-bib-0045] Rossi‐Stacconi, M. V. , Kaur, R. , Mazzoni, V. , Ometto, L. , Grassi, A. , Gottardello, A. , … Anfora, G. (2016). Multiple lines of evidence for reproductive winter diapause in the invasive pest *Drosophila suzukii:* Useful clues for control strategies. Journal of Pest Science, 89, 689–700. 10.1007/s10340-016-0753-8

[ece34928-bib-0046] Ryan, G. D. , Emiljanowicz, L. , Wilkinson, F. , Kornya, M. , & Newman, J. A. (2016). Thermal tolerances of the spotted‐wing Drosophila *Drosophila suzukii* (Diptera: Drosophilidae). Journal of Economic Entomology, 109, 746–752. 10.1093/jee/tow006 26880397

[ece34928-bib-0047] Schmidt, J. M. , Sebastian, P. , Wilder, S. M. , & Rypstra, A. L. (2012). The nutritional content of prey affects the foraging of a generalist arthropod predator. PLoS ONE, 7, e49223 10.1371/journal.pone.0049223 23145130 PMC3493534

[ece34928-bib-0048] Shearer, P. W. , West, J. D. , Walton, V. M. , Brown, P. H. , Svetec, N. , & Chiu, J. C. (2016). Seasonal cues induce phenotypic plasticity of *Drosophila suzukii* to enhance winter survival. BMC Ecology, 16, 11 10.1186/s12898-016-0070-3 27001084 PMC4802914

[ece34928-bib-0049] Silva‐Soares, N. F. , Nogueira‐Alves, A. , Beldade, P. , & Mirth, C. K. (2017). Adaptation to new nutritional environments: Larval performance, foraging decisions, and adult oviposition choices in *Drosophila suzukii* . BMC Ecology, 17, 21 10.1186/s12898-017-0131-2 28592264 PMC5463304

[ece34928-bib-0050] Simpson, S. J. , & Raubenheimer, D. (2011). The nature of nutrition: A unifying framework. Australian Journal of Zoology, 59, 350–368. 10.1071/zo11068

[ece34928-bib-0051] Sinclair, B. J. (2015). Linking energetics and overwintering in temperate insects. Journal of Thermal Biology, 54, 5–11. 10.1016/j.jtherbio.2014.07.007 26615721

[ece34928-bib-0052] Sinclair, B. J. , & Marshall, K. E. (2018). The many roles of fats in overwintering insects. Journal of Experimental Biology, 221 10.1242/jeb.161836 29514877

[ece34928-bib-0053] Stockton, D. G. , Wallingford, A. K. , & Loeb, G. (2018). Phenotypic plasticity promotes overwintering survival in a globally invasive crop pest, *Drosophila suzukii* . Insects, 9, e105. 30134571 10.3390/insects9030105 PMC6164111

[ece34928-bib-0054] Tochen, S. , Dalton, D. T. , Wiman, N. , Hamm, C. , Shearer, P. W. , & Walton, V. M. (2014). Temperature‐related development and population parameters for *Drosophila suzukii* (Diptera: Drosophilidae) on cherry and blueberry. Environmental Entomology, 43, 501–510. 10.1603/EN13200 24612968

[ece34928-bib-0055] Tochen, S. , Walton, V. M. , & Lee, J. C. (2016). Impact of floral feeding on adult *Drosophila suzukii* survival and nutrient status. Journal of Pest Science, 89(3), 793–802. 10.1007/s10340-016-0762-7

[ece34928-bib-0056] Toxopeus, J. , Jakobs, R. , Ferguson, L. V. , Gariepy, T. D. , & Sinclair, B. J. (2016). Reproductive arrest and stress resistance in winter‐acclimated *Drosophila suzukii* . Journal of Insect Physiology, 89, 37–51. 10.1016/j.jinsphys.2016.03.006 27039032

[ece34928-bib-0057] Venables, W. N. , & Ripley, B. D. (2002). Modern Applied Statistics with S, 4th ed. New York, NY: Springer.

[ece34928-bib-0058] Wallingford, A. K. , Lee, J. C. , & Loeb, G. M. (2016). The influence of temperature and photoperiod on the reproductive diapause and cold tolerance of spotted‐wing drosophila, *Drosophila suzukii* . Entomologia Experimentalis Et Applicata, 159, 327–337. 10.1111/eea.12443

[ece34928-bib-0059] Wallingford, A. K. , & Loeb, G. M. (2016). Developmental acclimation of *Drosophila suzukii* (Diptera: Drosophilidae) and its effect on diapause and winter stress tolerance. Environmental Entomology, 45, 1081–1089. 10.1093/ee/nvw088 27412194

[ece34928-bib-0060] Wallingford, A. K. , Rice, K. B. , Leskey, T. C. , & Loeb, G. (2018). Overwintering behavior of *Drosophila suzukii,* and potential springtime diets for egg maturation. Environmental Entomology, 47, 1266–1273. 10.1093/ee/nvy115 30124807

[ece34928-bib-0061] Walsh, D. B. , Bolda, M. P. , Goodhue, R. E. , Dreves, A. J. , Lee, J. , Bruck, D. J. , … Zalom, F. G. (2011). *Drosophila suzukii* (Diptera: Drosophilidae): Invasive pest of ripening soft fruit expanding its geographic range and damage potential. Journal of Integrated Pest Management, 2, G1–G7. 10.1603/ipm10010

[ece34928-bib-0062] Wickham, H. (2016). ggplot2: Elegant graphics for data analysis. New York, NY: Springer.

[ece34928-bib-0063] Wickham, H. , & Francois, R. (2016). dplyr: A grammar of data manipulation.

[ece34928-bib-0065] Wong, J. S. , Wallingford, A. K. , Loeb, G. M. , & Lee, J. C. (2018). Physiological status of *Drosophila suzukii* (Diptera: Drosophilidae) affects their response to attractive odours. Journal of Applied Entomology, 142, 473–482. 10.1111/jen.12497

[ece34928-bib-0066] Wong, J. S. , Cave, A. C. , Lightle, D. M. , Mahaffee, W. F. , Naranjo, S. E. , Wiman, N. G. , … Lee, J. C. (2018). *Drosophila suzukii* flight performance reduced by starvation but not affected by humidity. Journal of Pest Science, 91, 1269–1278. 10.1007/s10340-018-1013-x

[ece34928-bib-0067] Young, Y. , Buckiewicz, N. , & Long, T. A. F. (2018). Nutritional geometry and fitness consequences in *Drosophila suzukii,* the Spotted‐Wing Drosophila. Ecology and Evolution, 8, 2842–2851. 10.1002/ece3.3849 29531699 PMC5838031

