# Supplementary material for: Interactions among morphotype, nutrition, and temperature impact fitness of an invasive fly
Source: Ecol Evol. 2019 Feb 3;9(5):2615–28. doi: 10.1002/ece3.4928 (PMC6493778; doi:10.1002/ece3.4928)
Supplement: Supplementary file 1 [file ECE3-9-2615-s001.docx]

**Supplemental material Table 1.** Variance parameters for diet, temperature and morph on lifespan, fecundity, and pre-oviposition periods. Differences in lifespan were estimated using a Kaplan-Meier survival analysis, differences in fecundity and pre-oviposition periods were estimated using an analysis of variance (ANOVA) or t-test on transformed values.

| **Morph** | **Temperature (˚C)** | **Diet** | **Outcome variable** | **df** | **X^2^** | **p** |
| --- | --- | --- | --- | --- | --- | --- |
| Winter | 7 | Fixed effect | Lifespan (days) | 4 | 123 | < 0.01 |
| Winter | 9 | Fixed effect | Lifespan (days) | 4 | 130 | < 0.01 |
| Winter | 12 | Fixed effect | Lifespan (days) | 4 | 124 | < 0.01 |
| Winter | 14 | Fixed effect | Lifespan (days) | 4 | 172 | < 0.01 |
| Winter | 17 | Fixed effect | Lifespan (days) | 4 | 181 | < 0.01 |
| Summer | 9 | Fixed effect | Lifespan (days) | 3 | 48.9 | < 0.01 |
| Summer | 14 | Fixed effect | Lifespan (days) | 3 | 72 | < 0.01 |
| Summer | 17 | Fixed effect | Lifespan (days) | 3 | 33.7 | < 0.01 |
| Winter | Fixed effect | 0:0 | Lifespan (days) | 4 | 30.4 | < 0.01 |
| Winter | Fixed effect | 0:1 | Lifespan (days) | 4 | 21.4 | < 0.01 |
| Winter | Fixed effect | 1:4 | Lifespan (days) | 4 | 24.1 | < 0.01 |
| Winter | Fixed effect | 1:2 | Lifespan (days) | 4 | 9.11 | 0.06 |
| Winter | Fixed effect | 1:1 | Lifespan (days) | 4 | 17.8 | 0.02 |
| Summer | Fixed effect | 0:1 | Lifespan (days) | 3 | 12.3 | < 0.01 |
| Summer | Fixed effect | 1:4 | Lifespan (days) | 3 | 9 | 0.01 |
| Summer | Fixed effect | 1:2 | Lifespan (days) | 3 | 187.6 | < 0.01 |
| Summer | Fixed effect | 1:1 | Lifespan (days) | 3 | 5.3 | 0.06 |
| Fixed eff | 9 | 0:1 | Lifespan (days) | 1 | 7.7 | < 0.01 |
| Fixed eff | 9 | 1:4 | Lifespan (days) | 1 | 21.2 | < 0.01 |
| Fixed eff | 9 | 1:2 | Lifespan (days) | 1 | 2.2 | 0.1 |
| Fixed eff | 9 | 1:1 | Lifespan (days) | 1 | 0.6 | 0.4 |
| Fixed eff | 14 | 0:1 | Lifespan (days) | 1 | 3 | 0.08 |
| Fixed eff | 14 | 1:4 | Lifespan (days) | 1 | 3.5 | 0.06 |
| Fixed eff | 14 | 1:2 | Lifespan (days) | 1 | 5.6 | 0.02 |
| Fixed eff | 14 | 1:1 | Lifespan (days) | 1 | 0.1 | 0.8 |
| Fixed eff | 17 | 0:1 | Lifespan (days) | 1 | 10.1 | < 0.01 |
| Fixed eff | 17 | 1:4 | Lifespan (days) | 1 | 13.2 | < 0.01 |
| Fixed eff | 17 | 1:2 | Lifespan (days) | 1 | 5.6 | 0.02 |
| **Morph** | **Temperature (˚C)** | **Diet** | **Outcome variable** | **df** | **F** | **p** |
| Winter | 7 | Fixed effect | Fecundity (total eggs) | 1, 92 | 3.39 | 0.06 |
| Winter | 9 | Fixed effect | Fecundity (total eggs) | 1, 95 | 0.59 | 0.44 |
| Winter | 12 | Fixed effect | Fecundity (total eggs) | 1, 93 | 12.76 | < 0.01 |
| Winter | 14 | Fixed effect | Fecundity (total eggs) | 1, 96 | 12.62 | < 0.01 |
| Winter | 17 | Fixed effect | Fecundity (total eggs) | 1, 92 | 26.88 | < 0.01 |
| Summer | 9 | Fixed effect | Fecundity (total eggs) | 1, 59 | 0.53 | 0.46 |
| Summer | 14 | Fixed effect | Fecundity (total eggs) | 1, 60 | 8.67 | < 0.01 |
| Summer | 17 | Fixed effect | Fecundity (total eggs) | 1, 53 | 7.74 | < 0.01 |
| Winter | Fixed effect | 0:0 | Fecundity (total eggs) |  |  |  |
| Winter | Fixed effect | 0:1 | Fecundity (total eggs) | 1, 116 | 126.7 | < 0.01 |
| Winter | Fixed effect | 1:4 | Fecundity (total eggs) | 1, 118 | 109.6 | < 0.01 |
| Winter | Fixed effect | 1:2 | Fecundity (total eggs) | 1, 116 | 87.75 | < 0.01 |
| Winter | Fixed effect | 1:1 | Fecundity (total eggs) | 1, 120 | 5.37 | 0.02 |
| Summer | Fixed effect | 0:1 | Fecundity (total eggs) | 1, 38 | 9.835 | < 0.01 |
| Summer | Fixed effect | 1:4 | Fecundity (total eggs) | 1, 47 | 6.56 | 0.01 |
| Summer | Fixed effect | 1:2 | Fecundity (total eggs) | 1, 40 | 3.75 | 0.06 |
| Summer | Fixed effect | 1:1 | Fecundity (total eggs) | 1, 45 | 0.017 | 0.89 |
|  |  |  |  | **df** | **t** | **p** |
| Fixed eff | 9 | 0:1 | Fecundity (total eggs) | 16.92 | 1.71 | 0.1 |
| Fixed eff | 9 | 1:4 | Fecundity (total eggs) | 32.13 | -0.08 | 0.92 |
| Fixed eff | 9 | 1:2 | Fecundity (total eggs) | 21.99 | 1.3 | 0.2 |
| Fixed eff | 9 | 1:1 | Fecundity (total eggs) | 15.66 | 1.97 | 0.06 |
| Fixed eff | 14 | 0:1 | Fecundity (total eggs) | 31.89 | -0.36 | 0.71 |
| Fixed eff | 14 | 1:4 | Fecundity (total eggs) | 35.36 | -0.72 | 0.47 |
| Fixed eff | 14 | 1:2 | Fecundity (total eggs) | 29.84 | -1.1 | 0.27 |
| Fixed eff | 14 | 1:1 | Fecundity (total eggs) | 27.34 | 0.21 | 0.82 |
| Fixed eff | 17 | 0:1 | Fecundity (total eggs) | 18.13 | -2.13 | 0.04 |
| Fixed eff | 17 | 1:4 | Fecundity (total eggs) | 24.2 | -2.35 | 0.02 |
| Fixed eff | 17 | 1:2 | Fecundity (total eggs) | 25.12 | -2.26 | 0.03 |
| Fixed eff | 9 | 0:1 | Fecundity (total eggs) | 29.75 | -0.002 | 0.99 |
| **Morph** | **Temperature (˚C)** | **Diet** | **Outcome variable** | **df** | **F** | **p** |
| Winter | 7 | Fixed effect | Pre-oviposition period (days) | 1, 6 | 1.34 | 0.29 |
| Winter | 9 | Fixed effect | Pre-oviposition period (days) | 1, 18 | 3.82 | 0.06 |
| Winter | 12 | Fixed effect | Pre-oviposition period (days) | 1, 50 | 18.23 | < 0.01 |
| Winter | 14 | Fixed effect | Pre-oviposition period (days) | 1, 62 | 2.58 | 0.11 |
| Winter | 17 | Fixed effect | Pre-oviposition period (days) | 1, 68 | 3.94 | 0.06 |
| Summer | 9 | Fixed effect | Pre-oviposition period (days) | 1, 22 | 0.35 | 0.55 |
| Summer | 14 | Fixed effect | Pre-oviposition period (days) | 1, 41 | 24.02 | < 0.01 |
| Summer | 17 | Fixed effect | Pre-oviposition period (days) | 1, 31 | 3.24 | 0.08 |
| Winter | Fixed effect | 0:0 | Pre-oviposition period (days) |  |  |  |
| Winter | Fixed effect | 0:1 | Pre-oviposition period (days) | 1, 60 | 38.31 | < 0.01 |
| Winter | Fixed effect | 1:4 | Pre-oviposition period (days) | 1, 76 | 93.26 | < 0.01 |
| Winter | Fixed effect | 1:2 | Pre-oviposition period (days) | 1, 58 | 60.71 | < 0.01 |
| Winter | Fixed effect | 1:1 | Pre-oviposition period (days) | 1, 12 | 5.58 | 0.03 |
| Summer | Fixed effect | 0:1 | Pre-oviposition period (days) | 1, 25 | 0.57 | 0.45 |
| Summer | Fixed effect | 1:4 | Pre-oviposition period (days) | 1, 34 | 14.68 | < 0.01 |
| Summer | Fixed effect | 1:2 | Pre-oviposition period (days) | 1, 23 | 28.28 | < 0.01 |
| Summer | Fixed effect | 1:1 | Pre-oviposition period (days) | 1, 10 | 0.38 | 0.55 |
|  |  |  |  | **df** | **t** | **p** |
| Fixed eff | 9 | 0:1 | Pre-oviposition period (days) | 3 | -10.93 | < 0.01 |
| Fixed eff | 9 | 1:4 | Pre-oviposition period (days) | 14.79 | -1.62 | 0.12 |
| Fixed eff | 9 | 1:2 | Pre-oviposition period (days) | 5.89 | -1.21 | 0.26 |
| Fixed eff | 9 | 1:1 | Pre-oviposition period (days) | - | - | - |
| Fixed eff | 14 | 0:1 | Pre-oviposition period (days) | 20.58 | 0.93 | 0.36 |
| Fixed eff | 14 | 1:4 | Pre-oviposition period (days) | 28.52 | -3.12 | < 0.01 |
| Fixed eff | 14 | 1:2 | Pre-oviposition period (days) | 22.52 | -3.73 | < 0.01 |
| Fixed eff | 14 | 1:1 | Pre-oviposition period (days) | 3 | -4.18 | 0.02 |
| Fixed eff | 17 | 0:1 | Pre-oviposition period (days) | 14.14 | -0.2 | 0.84 |
| Fixed eff | 17 | 1:4 | Pre-oviposition period (days) | 19.21 | -2.23 | 0.03 |
| Fixed eff | 17 | 1:2 | Pre-oviposition period (days) | 12.06 | -2.05 | 0.06 |
| Fixed eff | 17 | 1:1 | Pre-oviposition period (days) | 3.99 | 0.22 | 0.83 |
