# Supplementary material for: Interactions among morphotype, nutrition, and temperature impact fitness of an invasive fly
Source: Ecol Evol. 2019 Feb 3;9(5):2615–28. doi: 10.1002/ece3.4928 (PMC6493778; doi:10.1002/ece3.4928)
Supplement: Supplementary file 2 [file ECE3-9-2615-s002.docx]

**Supplemental material Table S2.** General linear mixed model regression parameters on transformed values for the effect of diet and temperature and morph (model terms = diet*temperature*morph) on *D. suzukii* fecundity and pre-oviposition.

| **Outcome variable: fecundity.** | |  |  |
| --- | --- | --- | --- |
|  | **Estimate** | **Std. Error** | **t value** |
| (Intercept) | -1.462733 | 0.105617 | -13.849 |
| 9°C | 0.005579 | 0.147921 | 0.038 |
| 12°C | 0.95462 | 0.156315 | 6.107 |
| 14°C | 0.994536 | 0.150912 | 6.59 |
| 17°C | 1.295936 | 0.154372 | 8.395 |
| 0:0 | -0.203933 | 0.154372 | -1.321 |
| 1:4 | 0.040075 | 0.152577 | 0.263 |
| 1:2 | -0.203933 | 0.154372 | -1.321 |
| 1:1 | -0.203933 | 0.150912 | -1.351 |
| Summer morph (SM) | -0.131038 | 0.195008 | -0.672 |
| 9°C * 0:0 | -0.005579 | 0.223718 | -0.025 |
| 12°C * 0:0 | -0.95462 | 0.225951 | -4.225 |
| 14°C * 0:0 | -0.994536 | 0.222248 | -4.475 |
| 17°C * 0:0 | -1.295936 | 0.226239 | -5.728 |
| 9°C * 1:4 | 0.516896 | 0.213601 | 2.42 |
| 12°C * 1:4 | -0.107231 | 0.22189 | -0.483 |
| 14°C * 1:4 | 0.276948 | 0.214603 | 1.291 |
| 17°C * 1:4 | -0.020076 | 0.216059 | -0.093 |
| 9°C * 1:2 | 0.181739 | 0.216059 | 0.841 |
| 12°C * 1:2 | 0.077117 | 0.219694 | 0.351 |
| 14°C * 1:2 | 0.301672 | 0.216957 | 1.39 |
| 17°C * 1:2 | 0.108159 | 0.219378 | 0.493 |
| 9°C * 1:1 | 0.040958 | 0.212415 | 0.193 |
| 12°C * 1:1 | -0.759887 | 0.215366 | -3.528 |
| 14°C * 1:1 | -0.731175 | 0.213422 | -3.426 |
| 17°C * 1:1 | -0.974759 | 0.219378 | -4.443 |
| 9°C * SM | 0.442462 | 0.25951 | 1.705 |
| 14°C * SM | 0.243969 | 0.263756 | 0.925 |
| 1:4 * SM | -0.304613 | 0.25951 | -1.174 |
| 1:2 * SM | -0.329159 | 0.263756 | -1.248 |
| 1:1 * SM | 0.161384 | 0.26324 | 0.613 |
| 9°C * 1:4 * SM | 0.030289 | 0.354558 | 0.085 |
| 14°C * 1:4 *SM | 0.167439 | 0.354305 | 0.473 |
| 9°C * 1:2 * SM | 0.359402 | 0.361841 | 0.993 |
| 14°C * 1:2 * SM | 0.09402 | 0.364205 | 0.258 |
| 9°C * 1:1 * SM | -0.097765 | 0.357298 | -0.274 |
| 17°C * 1:1 * SM | -0.271519 | 0.359748 | -0.755 |

| **Outcome variable: pre-oviposition period** | | |  |
| --- | --- | --- | --- |
|  | **Estimate** | **Std. Error** | **t value** |
| (Intercept) | -0.27014 | 0.120367 | -2.244 |
| 9°C | 0.190611 | 0.170225 | 1.12 |
| 12°C | 0.100737 | 0.134575 | 0.749 |
| 14°C | -0.127258 | 0.133071 | -0.956 |
| 17°C | -0.296032 | 0.131856 | -2.245 |
| 1:4 | 0.105285 | 0.170225 | 0.619 |
| 1:2 | -0.045121 | 0.075215 | -0.6 |
| 1:1 | -0.29402 | 0.120367 | -2.443 |
| SM | -0.058286 | 0.093236 | -0.625 |
| 9°C * 1:4 | -0.268421 | 0.21976 | -1.221 |
| 12°C * 1:4 | -0.187133 | 0.190951 | -0.98 |
| 14°C * 1:4 | -0.131351 | 0.186322 | -0.705 |
| 17°C * 1:4 | -0.226869 | 0.185173 | -1.225 |
| 9°C * 1:2 | 0.002777 | 0.198654 | 0.014 |
| 12°C * 1:2 | -0.07248 | 0.112643 | -0.643 |
| 14°C * 1:2 | -0.024092 | 0.109211 | -0.221 |
| 9°C * 1:1 | 0.048998 | 0.294839 | 0.166 |
| 12°C * 1:1 | -0.078378 | 0.180551 | -0.434 |
| 14°C * 1:1 | 0.254245 | 0.179433 | 1.417 |
| 9°C * SM | -0.661322 | 0.186472 | -3.546 |
| 14°C * SM | 0.129317 | 0.129391 | 0.999 |
| 1:4 * SM | -0.169611 | 0.130012 | -1.305 |
| 1:2 * SM | -0.215934 | 0.133761 | -1.614 |
| 1:1 * SM | 0.023015 | 0.186472 | 0.123 |
| 9°C * 1:4 * SM | 0.725748 | 0.232918 | 3.116 |
| 14°C * 1:4 *SM | -0.178782 | 0.175729 | -1.017 |
| 9°C * 1:2 * SM | 0.841267 | 0.273641 | 3.074 |
| 14°C * 1:2 * SM | -0.199983 | 0.185093 | -1.08 |
| 9°C * 1:1 * SM | 0.316064 | 0.361102 | 0.875 |
| 17°C * 1:1 * SM | -0.45601 | 0.276816 | -1.647 |
